# The application of impantable sensors in the musculoskeletal system: a review

**DOI:** 10.3389/fbioe.2024.1270237

**Published:** 2024-01-24

**Authors:** Jinzuo Wang, Jian Chu, Jinhui Song, Zhonghai Li

**Affiliations:** ^1^ Department of Orthopaedics, First Affiliated Hospital of Dalian Medical University, Dalian, China; ^2^ Key Laboratory of Molecular Mechanism for Repair and Remodeling of Orthopaedic Diseases, Dalian, Liaoning, China; ^3^ Department of Engineering Mechanics, Dalian University of Technology, Dalian, China

**Keywords:** implantable sensor, flexible material, joint, hip, knee, spine, fracture

## Abstract

As the population ages and the incidence of traumatic events rises, there is a growing trend toward the implantation of devices to replace damaged or degenerated tissues in the body. In orthopedic applications, some implants are equipped with sensors to measure internal data and monitor the status of the implant. In recent years, several multi-functional implants have been developed that the clinician can externally control using a smart device. Experts anticipate that these versatile implants could pave the way for the next-generation of technological advancements. This paper provides an introduction to implantable sensors and is structured into three parts. The first section categorizes existing implantable sensors based on their working principles and provides detailed illustrations with examples. The second section introduces the most common materials used in implantable sensors, divided into rigid and flexible materials according to their properties. The third section is the focal point of this article, with implantable orthopedic sensors being classified as joint, spine, or fracture, based on different practical scenarios. The aim of this review is to introduce various implantable orthopedic sensors, compare their different characteristics, and outline the future direction of their development and application.

## 1 Introduction

As technology continues to advance, significant progress has been made in the improvement of medical diagnosis and treatment. The increasing need for precision in diagnosing and treating diseases is emphasized by the prevalence of degenerative conditions in orthopedics, including osteoarthritis, disc herniation, and spinal stenosis. The diagnosis of degenerative disease involves a combination of imaging techniques and the subjective perception reported by the patient. End-stage degenerative diseases can often lead to disability, necessitating surgical intervention. Most orthopedic procedures require the use of implants for the replacement of normal anatomical structures. In 2021, the global implant market reached USD 157.97 billion, with orthopedics comprising 37% of this total (Veletić et al., 2022). Such surgery can provide pain relief and restore function, thus improving quality of life (Wijayaratna et al., 2021); however, the placement of an implant does not mark the conclusion of the treatment process. Post-surgical monitoring of implants is essential because of potential complications including infection, inflammation, and loosening. These issues not only contribute to the failure of the implant but can also necessitate a secondary procedure, posing serious risks including potential threats to the patient’s life. Early detection of issues enables timely intervention, helping to prevent the occurrence of adverse events.

Currently, the monitoring of implants primarily involves imaging and laboratory examinations. Imaging techniques, such as X-ray, CT and MRI, provide insights into the implant’s condition by observing its position and the morphology of the surrounding soft tissue. Laboratory examination assesses the presence of inflammation in the body by analyzing the peripheral blood and other tissues; however, these examinations are less specific. Imaging examination depends on the expertise of experienced doctors and often lack quantitative indicators. Moreover, the repeated nature of the examination process, coupled with the potential adverse effects on the body, underscores the need for a shift towards *in vivo* sensor technology.

In simple terms, a sensor is a detection device, typically comprising a sensing element and a conversion element. Its primary function is to measure information, enabling users to access and interpret the gathered data. Sensor data can be converted into an electrical signal or other desired output form to meet the requirements for information transmission, processing, storage, display, and recording. Sensors play an important role in various aspect of life. In 1958, a 43-year-old Swedish man received the first implantable sensor—a cardiac pacemaker (Nicholls, 2007). This device uses a sensor to monitor heart rate, sending out corrective pulses when the heart rate falls below a predetermined parameter. Subsequently, a wide range of implantable sensors have been employed for diagnostic and therapeutic purposes in cardiology, neurology, orthopedics, and gastroenterology (Schulman et al., 2004; Donelli et al., 2007; Ledet et al., 2012).

Implantable sensors are typically integrated into what are commonly referred to as smart implants. These smart implants not only offer the therapeutic functions of regular implants but also incorporate diagnostic capabilities. Smart implants can collect data from the patient’s body, thus enabling personalized medicine (Burny et al., 2000). Therefore, implants have been developed to provide multi-functional capabilities across various bioapplications. In the field of orthopedics, four methodologies have been proposed for designing implant technologies: non-instrumented passive implants, non-instrumented active implants, instrumented passive implants, and instrumented active implants. As a diagnostic tool, the greatest advantage of implantable sensor technology is that it can provide data not available through any other means. These data are objective, quantitative, and real-time, serving as a prompt for physicians to take proactive steps in treatment, detect complications early, reduce recovery time, and optimize patient recovery. Research based on implantable sensors makes an important contribution to understanding the onset and progression of diseases, as well as post-operative recovery. Therefore, we have undertaken a comprehensive review of the research progress in the application of implantable sensors for the musculoskeletal system. Through this review, we aim to provide medical and engineering experts with valuable insights into the development of implantable sensors for clinical orthopedic applications.

## 2 Sensor classification

When developing implantable sensors, key considerations include accuracy, size, and cost. Because the measurement requirements differ, the components of the sensor differ as well. Sensors are classified into five categories based on their working principles and operations: electric sensors, piezoelectric sensors, optical sensors, thermal sensors, and electrochemical sensors.

### 2.1 Electric sensors

Electrical sensors are considered the simplest and most commonly used. They operate on the principle that their resistance, capacitance, and inductance change in response to a physical stimulus. Electrical sensors can be further divided into resistive, capacitive, and inductive sensors based on their electronic components.

#### 2.1.1 Resistive sensors

Resistive sensors operate on the principle that the resistance in a circuit changes in response to variations in a physical quantity. Featuring a simple construction, durability, wide dynamic range, and low cost, they are suitable for applications such as temperature and strain sensing. The most common resistive sensors include resistance temperature detectors (RTDs), thermistors, and strain gauges. The key feature enabling temperature measurement in RTDs and thermistors is their high linearity between resistance and temperature. Typically, RTDs have a resistance temperature coefficient value of 3.33 × 10^−3^/°C with an accuracy of ± 0.2°C, over a measurement range of 20°C–100°C (Kim et al., 2020b). Therefore, RTDs and thermistors are used to measure implant temperatures, with a temperature increase indicating a possible implant infection (Yu et al., 2017). Strain gauges are usually attached to the surface of a metal elastomer. As the elastomer undergoes deformation from an external force, the strain is transmitted through the adhesive to the resistive strain gauge, resulting in a change in the resistance value of the strain gauge (Zhao et al., 2020). Strain gauges are the most widely used sensors for measuring a variety of complex forces *in vivo* across different scenarios. These applications include forces in joints, displacement during fracture healing, forces acting on dental implant prostheses, and bladder pressure (Kim et al., 2012; D'Lima et al., 2013; Cho et al., 2014; Pelham et al., 2017). Fukase et al. (2022) developed a smart bone plate to wirelessly monitor healing utilizing electrical impedance spectroscopy to provide real-time data on tissue composition within the fracture callus. The sensor is wireless and can take readings up to 3 m from the implanted bone for up to 8 weeks.

#### 2.1.2 Capacitive sensors

The capacitor is the key electronic component in a capacitive sensor. Typically, a capacitor comprises two parallel conductive plates at the top and bottom, with a dielectric material positioned in between. When the measured physical quantity changes, alterations occur in the distance between the electrodes, their overlapping area, or the dielectric properties of the material (Qin et al., 2021). Selecting materials with a low temperature coefficient for electrodes in capacitive sensor construction ensures they are almost temperature independent (Sander et al., 1980). Moreover, capacitive sensors offer advantages such as simple device structure, low power consumption, versatile applications, rapid dynamic response, and high durability (Sun et al., 2014; Guo et al., 2019; Liu et al., 2020). They are commonly employed for level, strain, force, pressure, and humidity monitoring. Because of their high sensitivity, capacitive sensors can detect small force magnitudes, proving valuable in applications such as the histomorphometric evaluation of dental implants, and tracking bone growth surrounding them over time (D’Lima et al., 2013). In musculoskeletal system, capacitive sensors are often used to monitor implant loosening and fracture healing. Sorriento et al. (2021) proposed a system based on capacitive sensor technology, able to measure, quantitatively, the relative pins displacements in bone fractures treated with external fixators with a resolution of 0.5 mm and 0.5°.

#### 2.1.3 Inductive sensors

Inductive sensors are typically composed of a magnetic core with an air gap and a coil (Veletić et al., 2022). The magnetic resistance is determined by the length of the air gap, with longer gaps resulting in higher magnetic resistance. Even a very small change in the air gap size can cause a significant change in magnetic resistance. Because of their high sensitivity, inductive sensors are frequently used to measure small changes such as displacement and strain and are often integrated into an inductor-capacitor circuit (Chen et al., 2014b; Burton et al., 2021). Bennett et al. (2021) developed a sensor to detect the depth of a locking pin in the shuttle lock of a transtibial socket and to monitor the small motions between ratchet clicks during ambulation. The sensor demonstrated a root mean square error of 0.21% of the full-scale output and it is sufficiently accurate. Additionally, these sensors can be used for wireless communication and power transmission, making them particularly promising for the remote monitoring of implants.

### 2.2 Piezoelectric sensors

The operating principle of piezoelectric sensors can be categorized into direct or reverse piezoelectricity. The piezoelectric effect occurs when a piezoelectric material is deformed, generating an electrical charge. The reverse piezoelectric effect involves the deformation of the material when a voltage is applied. Therefore, the biggest advantage of piezoelectric sensors is their ability to be self-powered. Piezoelectric sensors are commonly used in knee joints, serving as a self-powered sensor that can measure contact forces within the knee joint (Vassiliadis and Matsouka, 2018). Safaei et al. (2020) proposed an instrumented knee implant design with six piezoelectric transducers embedded in the tibial bearing. The experimental results show that the instrumented knee bearing is able to accurately measure the compartmental force quantities with a maximum error of 2.6% of the peak axial load, and the contact point locations with a maximum error of less than 1 mm. Piezoelectric sensors are ideal for application as a biosensor, to facilitate the measurement of various microorganisms and biomolecules (Montanaro et al., 2011; Pohanka, 2018). Piezoelectric sensors are also used in the development of electronic skins capable of monitoring pulse and temperature. Additionally, they are integrated into cochlear implants to monitor sound vibrations (Ilik et al., 2018; Vassiliadis and Matsouka, 2018).

### 2.3 Optical sensors

Optical sensors can detect changes in the optical properties of its environment through a photodetector and are commonly employed in eye implants (Choi et al., 2017). Because of their high sensitivity and electrical passivity, they are also used to determine dental bite forces, with a relative error rate of less than 7% (Elsarnagawy and El-Wakad, 2008). Advantages also include the ability to withstand electromagnetic interference during head and neck MRI procedures. Additionally, their chemical inertness helps to prevent the induction of inflammatory responses and corneal edema (Lee et al., 2017). Optical sensors are rarely used in musculoskeletal systems. Cai et al. (2021) developed a new class of wireless battery-free devices, named osseosurface electronics, which feature soft mechanics, ultra-thin form factor and miniaturized multimodal biointerfaces comprised of sensors and optoelectronics directly adhered to the surface of the bone. The experiments demonstrated its potential as a fully implantable device.

### 2.4 Thermal and thermoelectric sensors

Thermistors represent the most common application of thermal sensors, where the resistance varies in response to temperature changes (Ramanathan and Danielsson, 2001). Benca et al. (2021) used thermal sensors to measure the amount of heat generated during drilling. The result showed that drilling caused a temperature increase of <2.5°C and the mean increase in temperature during thread tapping and implant insertion was <5.0°C. Inflammation and infection often cause a small increase in temperature, making thermistors a common choice for diagnostic purposes (Kim et al., 2020b). They have been employed in dentistry and orthopedics to determine if an implant is infected (Kim et al., 2020a; Benca et al., 2021). Thermoelectric sensors work by changing the voltage produced in response to a temperature difference between two electrodes (Ramanathan and Danielsson, 2001). A widely used type of thermoelectric sensor is the thermocouple-based sensor, which provides simple measurements across a broad temperature range (Bhatia et al., 2010). However, it is important to note that thermocouple-based sensors have low sensitivity, which requires additional conditioning.

### 2.5 Electrochemical sensors

Electrochemical sensors are based on the chemical reaction method and are typically classified into conductometric, potentiometric, and amperometric sensors. Conductivity sensors can modulate their resistance in response to a chemical reactions. Their high sensitivity makes them suitable for monitoring electrolytes and metabolites (Li et al., 2007). Potentiometric sensors generate voltage through electron exchange between the sensing element and the solution. Therefore, they often find application in monitoring solution pH and the metabolites of degradable materials, such as magnesium. A decrease in pH is regarded as an early indicator of implant infection, making it a valuable parameter for monitoring the status of the implant (Waizy et al., 2013). In potentiometric sensors, the analytical information obtained from the biorecognition process is converted into electric potential, whereas in amperometric sensors, the constant potential current associated with reduction or oxidation of an electroactive species is monitored (Singh et al., 2016). Therefore, they are extensively employed in disease diagnostics for the detection of suitable marker proteins, antibodies, DNA sequences, or cells (Singh et al., 2016). In addition to monitoring implant infections, electrochemical sensors are rarely used in the musculoskeletal system.

## 3 Implantable sensor materials

Sensors are typically composed of conductors, semiconductors, and dielectrics. Additionally, for implantable devices, substrates and encapsulation are usually required for bonding with the tissue (Singh et al., 2020). The selection of materials is a critical consideration in the construction of implantable sensors, with the choice varying based on the specific application, such as pressure monitoring, flow monitoring, strain detection, and chemical sensing (DiMarco, 2003; Wilson and Dorman, 2008). Implant systems are required to function within the human body for extended periods ranging from months to years; therefore, one of the most important considerations is the biocompatibility of the material (Onuki et al., 2008). Recent advances in microelectromechanical systems (MEMS) and nanoelectromechanical systems (NEMS) have enabled the miniaturization of functional electronic components. This progress has fueled a growing research trend towards increasingly compact sensors. Conventional rigid materials are no longer sufficient for implantable sensors. Flexible materials are at the forefront of emerging advancements in implantable materials, valued for their ability to conform to curved and soft tissues. The advantages and applications of frequently used implantable sensor materials are detailed in Table 1.

**TABLE 1:** Common materials of implantable sensors.

	Materials	Advantages	Application
Rigid material	Si and SiO_2_	Ease of micromachining with high resolution	Structural material and bulk substrate
	Si_3_N_4_	Thermally stable, non-toxic and biocompatible	Dielectric layer and insulation layer
	Metals and oxides	Favorable mechanical properties and biocompatible	Substrate
Flexible material	PDMS	Low Young’s modulus, imperviousness to fluids, high dielectric strength, low chemical reactivity and biocompatibility	Substrate
	Medical grade silicone	High tear strength, outstanding elasticity over a wide temperature range	Substrate
	Parylene-C	Biocompatibility, chemical and biological inertness, good barrier properties with low water permeability and absorption	Substrate and encapsulation
	PI	Thermal and chemical stabilities, low dissipation factors, and low dielectric constants	Passivation or insulation material and substrate layer
	PVDF	Piezoelectric response, thermal and chemical stability	Piezoelectric film
	LCP	Lower moisture absorption rate and no electrical degradation	Substrate and encapsulation material

PDMS, polydimethylsiloxane; PI, polyimide; PVDF, polyvinylidene fluoride; LCP, liquid crystal polymer.

### 3.1 Rigid materials

Common rigid materials include silicon (Si), glass silicon dioxide (SiO_2_), silicon nitride (Si_3_N_4_), and metal/metallic oxides. These materials exhibit high Young’s moduli, hardness, and temperature processing limits, as well as low gas permeability.

#### 3.1.1 Silicon and silicon dioxide

Si is commonly used as a structural material or bulk substrate in implantable sensors. SiO_2_ is a rigid material that is also widely used in implantable sensors, particularly for blood pressure measurements (Liang et al., 2011; Ye et al., 2011; Murphy et al., 2013). Different types of sensors are made by etching various patterns on the surface of Si. Although Si is inherently hard and stiff, it can be used as a thin membrane in implantable pressure sensors, which convert physiological pressure into a measurable capacitive change or piezoelectric signal (Yoon et al., 2004).

#### 3.1.2 Biological ceramic

Si_3_N_4_ is a thermally stable, non-toxic, and biocompatible ceramic material, making it more suitable than other ceramic materials for use in orthopedic implants. In addition, Si_3_N_4_ can be deposited on both sides of a silicon wafer to serve as a hard mask during etching processes, which is particularly useful in the design of implantable sensors for cerebrospinal fluid (CSF) flow monitoring (Raj et al., 2015; Apigo et al., 2016; Apigo et al., 2017). This pressure sensor is capable of detecting CSF pressure, hydrostatic pressure, and flow by measuring the deflection of a flexible membrane. However, it exhibits relatively low sensitivity and is primarily suitable for measuring slow-moving fluids in clinical contexts, such as for CSF.

#### 3.1.3 Metals and oxides

Metallic substrates are also used in the fabrication of implantable sensors, with stainless steel being a common choice. Chen et al. (2017) developed a capacitive pressure sensor on a stainless steel chip, designed for seamless integration with inductive stents using micro welding. The use of stainless steel as the substrate improved the sensor’s reliability. In addition, various metals, including Zn, Cu, Al, Au, and Ag are used as conductive materials in the construction of implantable sensors (Heller, 2006; Soebadi et al., 2020; Yang et al., 2020; Lee et al., 2022).

The application of rigid materials in implantable sensors poses many challenges. Firstly, the majority of rigid materials are not biocompatible, necessitating additional encapsulation materials to reduce the risk of harming the body. The bonding of the encapsulation material to the sensor increases the complexity of the sensor manufacturing process. Secondly, the sharp edges of hard materials may pose a risk of injuring soft tissues during implantation into the body.

### 3.2 Flexible materials

The evolution of polymer materials has led to the production of various biocompatible polymers, such as polydimethylsiloxane (PDMS), medical grade silicone, parylene-C, polyimide (PI), polyvinylidene fluoride (PVDF), and liquid crystal polymers (LCP). These materials are typically soft, lightweight, and low cost and are widely used in the manufacture of substrates, sensing elements, and encapsulations for implantable sensors.

#### 3.2.1 Polydimethylsiloxane

PDMS is a type of silicone elastomer with a low Young’s modulus, fluid impermeability, high dielectric strength, low chemical reactivity, and excellent biocompatibility (Lee and Choi, 2008). PDMS is used in sensitive capacitive sensors designed for measuring pressure and oxygen levels in the heart and blood vessels, as well as for monitoring nerve tissue health (Chiang et al., 2007; Koley et al., 2009). The low Young’s modulus of PDMS makes it suitable for use as a flexible substrate for sensors. Even under bending or stretching, PDMS maintains its structural integrity without undergoing permanent deformation. Chen et al. (2022) proposed a fabrication method for a double-layer piezoresistive pressure sensor, aiming to achieve a wide sensing range and high sensitivity. A conductive pathway was constructed on PDMS films and the resulting sensor exhibited a high sensitivity of 2.6 kPa^−1^ within a wide linear range of 0–30 kPa, along with fast response and recovery times of 40/40 ms. It demonstrated excellent reproducibility and was successfully applied to the real-time detection of radial artery heart rate, limb movement, handwriting, and vocal cord vocalization.

#### 3.2.2 Medical-grade silicone

Medical-grade silicone, a type of silicone elastomer approved for use in biomedical implants by the Food and Drug Administration, has excellent biocompatibility. In comparison to PDMS, it has high tear strength and superior elasticity across a broad temperature range. Medical-grade silicone is an ideal candidate for substrate materials in implantable sensors. This is demonstrated in the fabrication of soft contact lens sensors, where resonance circuits are embedded within layers of medical-grade silicone for continuous intraocular pressure monitoring (Chen et al., 2014a; Farandos et al., 2015). In addition, medical-grade silicone can be used to make strain gauge housing strips for measuring blood pressure through the deformation of blood vessels (Bingger et al., 2012). It has a similar Young’s modulus to that of blood vessels which minimizes interference when wrapped around the vessel.

#### 3.2.3 Parylene-C

Parylene-C is widely used as a substrate or encapsulation material for implantable sensors because of its biocompatibility, chemical and biological inertness, effective barrier properties with low water permeability and absorption, and its functionality as an electrical insulator. The electrochemical impedance of microbubbles can be measured through the electrolysis of a platinum electrode in contact with the Parylene-C surface. This unique property enables the use of Parylene-C in the preparation of standard sensors for monitoring hydrocephalus treatment and shunt performance (Yu et al., 2015; Kim et al., 2016). Using Parylene-C as a foundation, Yao et al. (2020) designed an ultrathin, flexible, waterproof, and robust micro-nano composite coating for encapsulating an implantable pressure sensor. Experimental results demonstrate its functional performance in both simulated body fluid environments and animal experiments.

#### 3.2.4 Polyimide

PI is a type of polymer composed of imide monomers and is known for its high heat resistance and glass transition temperatures, remaining stable up to 440°C (Liaw et al., 2012). Its biocompatibility was tested using standard methods according to International Organization for Standardization 10,993, and the results indicated that the electrodes were non-cytotoxic, non-mutagenic, non-clastogenic, and non-hemolytic (Kullmann et al., 2022). PIs are widely used in sensors as passivation or insulation materials and substrate layers because of their excellent thermal and chemical stabilities, low dissipation factors, and low dielectric constants. PIs are commonly used as thin films, serving as structural components in implantable sensors. In a wireless intraocular pressure sensor, the copper inductor pattern is deposited on top of the PI membrane (Kang et al., 2013). A thin PI film is also used as the diaphragm in capacitive pressure transducers for implantable cardiovascular applications (Hasenkamp et al., 2012). Zhu et al. (2022) introduced a PI-based film bulk acoustic resonator humidity sensor with high sensitivity and stability. This sensor was applied for the first time in the real-time monitoring of human respiration.

#### 3.2.5 Polyvinylidene fluoride

PVDF and its copolymer, polyvinylidene fluoride-trifluoroethylene (PVDF-TrFE), have a wide range of applications because of their piezoelectric response, thermal resistance, and chemical stability. PVDF is a thermoplastic semi-crystalline polymer which, in more of being cheap and easy-to-process, exhibits attractive electroactive properties (Song et al., 2022a; Li et al., 2022; Yuan et al., 2022). PVDF scaffolds for tissue engineering were proven to have good biocompatibility (Kitsara et al., 2022). Their piezoelectric properties make them valuable components in acoustic sensors. PVDF-TrEE is a lightweight material and can sustain higher strains (Zhang et al., 2002). The PVDF-TrFE diaphragm exhibits high linearity in response to small pressure changes, high sensitivity, and insensitivity to ambient temperature changes (Li et al., 2010). This diaphragm can be integrated with a catheter for intravascular measurements and used for monitoring heartbeat and respiration by measuring deformation of the chest wall (Chiu et al., 2013; Sharma et al., 2013).

#### 3.2.6 Liquid crystal polymer

LCP belongs to the family of aromatic polymers. LCP has been widely employed as an insulating and substrate material in the implantable devices due to its biocompatibility and low moisture absorption rate (<0.04%) compared to other polymers such as PI, parylene-C, or silicon elastomer (Kim et al., 2020a; Yun et al., 2022; Ahn et al., 2023). The use of LCP encapsulation provides long-term reliability without electrical degradation. *In vivo* studies demonstrate minimal fibrous encapsulation and inflammation in LCP-packaged devices (Chow et al., 2010). Sattayasoonthorn et al. (2019) developed a biocompatible, miniature, and implantable wireless intracranial pressure monitoring device. The device was primarily fabricated using LCP through standard microelectromechanical system procedures. The results demonstrated the sensor’s capability to perform intracranial pressure measurements in a humid environment within the range of 0–30 mmHg.

## 4 Implantable sensors for the musculoskeletal system

The growth and repair of musculoskeletal tissues are intricately linked to stresses and strains. Therefore, gaining insights into the forces and deformations occurring within bones and joints contributes to an enhanced understanding of diseases. While certain sensors are already deployed in clinical settings to measure joint and spine forces *in vivo*, the majority of implantable sensors remain in the preclinical stage. This section will provide an overview of the latest research advancements in orthopedic implantable sensors, focusing on three key aspects: joints, spine, and fractures.

### 4.1 Joints

Joint replacement is an important treatment strategy for severe osteoarthritis, with hip and knee replacement surgeries being the most common procedures. Each year, more than one million hip and knee replacements are performed worldwide, and the number continues to increase (Ferguson et al., 2018; Price et al., 2018). Given the potential risks related to implant wear, loosening, and infection following joint replacement, real-time monitoring of the prosthesis to gain insights into the stresses within the joint is essential for post-operative rehabilitation. Therefore, specialized implantable sensors have been developed to address these needs.

#### 4.1.1 Hip

In 1966, the first implantable sensor was introduced for application in hip implants. Rydell (1966) placed a strain gauge on the neck of the metal prosthesis to measure the forces acting on a mobile patient’s femur prosthesis. The system involved connecting one end of a wire to the prosthesis and the other end to an external data recorder through a percutaneous connection. While not a fully implantable sensor, this research marked a significant milestone. It was not until 1974 that a truly wireless implantable sensor was developed. Carlson et al. (1974b) placed 14 pressure sensors on the hemispheres of a prosthesis to measure pressure on the surface of the hip cartilage. Subsequent applications of similar sensors have extended to the measurement of hip pressure during daily activities using remote sensing or battery power (English and Kilvington, 1979; Davy et al., 1988; Bergmann et al., 2004; Damm et al., 2010).

Post-operative monitoring extended from 12 days to 36 months (Carlson et al., 1974a; Hodge et al., 1989; Cristofolini et al., 2000; Wei et al., 2022). During extended walks, hip prostheses experienced heat generation because of friction. Prolonged exposure to intra-articular heat may inhibit the growth of periarticular cells, potentially resulting in bone resorption or implant loosening (Pritchett, 2011). Bergmann et al. (2012) conducted a clinical trial involving 100 individuals, during which a temperature sensor was placed in the neck of the prothesis (Force sensor and IMU sensor). The sensor measured implant temperature within the range of 20°C–58°C, with an accuracy of 0.1°C. Some implants can incorporate dual sensors for simultaneous force and temperature measurement (Graichen et al., 1999). In addition, Lange et al. (2021) designed a piezoelectric sensor-based hip implant powered by the pressure exerted within the joint. This innovative approach could pave the way for future developments in implantable sensor design.

In addition to measuring temperature, force, and pressure, implantable sensors play an important role in monitoring the stability of implants by detecting any signs of loosening (Ruther et al., 2011). Heilemann et al. (2020) developed a pin-socket sensor system capable of monitoring micro-movements within a range of 59 ± 2 μm to 222 ± 5 μm on the implant surface. However, the drawback of this approach is that many holes need to be drilled in the implant surface to mount the sensor, which may cause structural damage to the implant. Mohammadbagherpoor et al. (2020) designed a wireless inductive proximity sensor system for detecting early implant loosening (Inductive-capacitive passive sensors). Its key advantage lies in the high sensitivity of the inductive sensor, which is capable of monitoring loosening at a distance of 20 mm with a resolution of 100 μm. de Sousa et al. (2021) designed a surface capacitive architecture sensing system to detect bone implant loosening, achieving favorable outcomes in both *in vitro* experiments and numerical simulations. Another study found that extracorporeal informatic systems enable continuous patient monitoring through cosurface capacitive networks, with or without hydroxyapatite-based layers (Peres et al., 2022). This could represent a significant advancement in the design of multi-functional smart implants. Additionally, the team proposed a new cosurface-based capacitive system designed to deliver controllable and personalized electric field stimuli to target tissues (Soares Dos Santos et al., 2016; Bernardo et al., 2019; Ramos and Soares Dos Santos, 2023). This system not only enhanced the predictability of sensing bone-implant fixation states but also delivered personalized stimulation to peri-implant tissues (de Sousa et al., 2021; Ramos and Soares Dos Santos, 2023).

**FIGURE 1:**
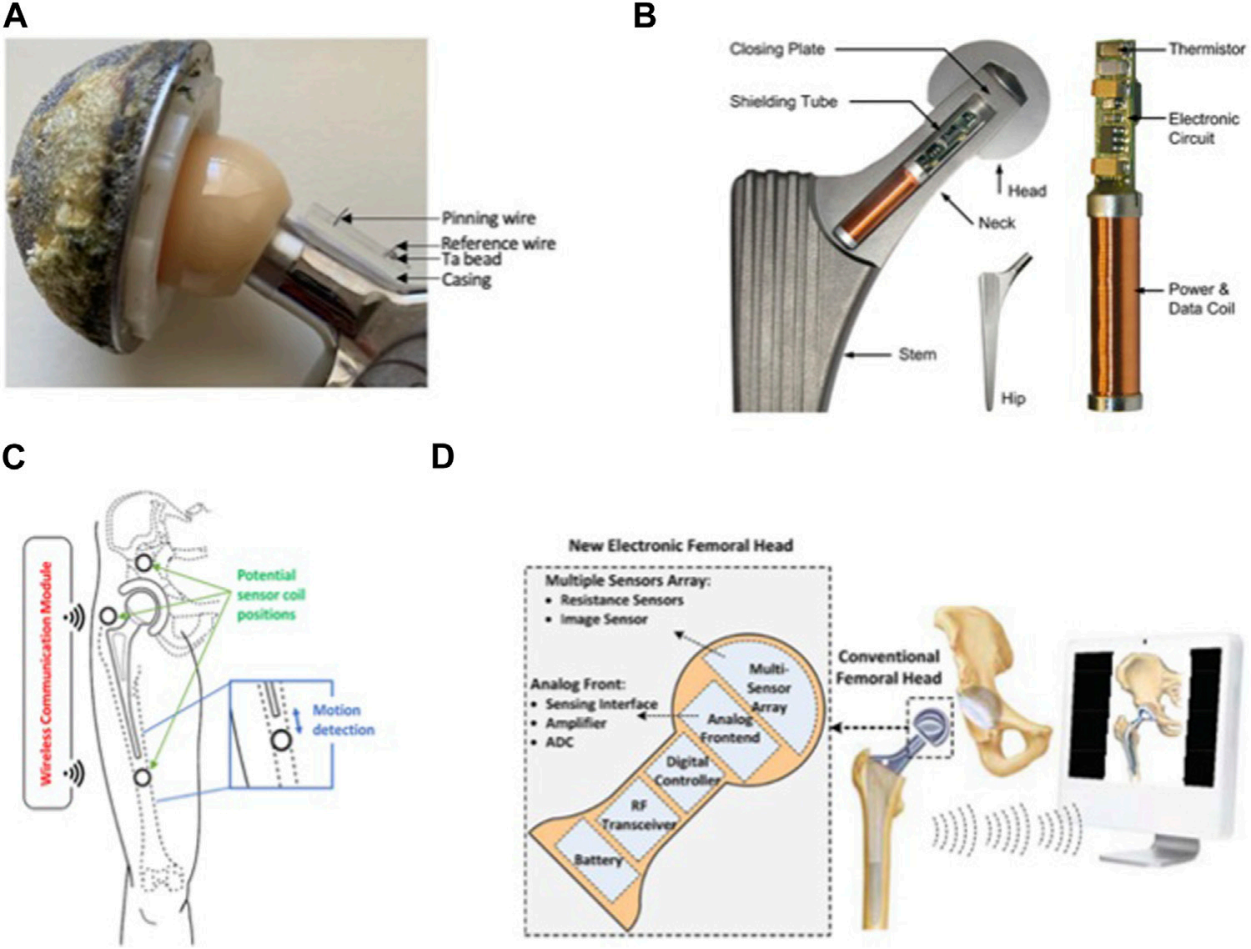
Implantable sensors for the hip: **(A)** An implantable sensor developed for non-invasive early detection of synovial fluid pH. Reproduced with permission from (Wijayaratna et al., 2021). **(B)** A hip endoprosthesis equipped with sensors to measure joint contact forces and temperature distribution along the entire length of the titanium implant. Reproduced with permission from (Bergmann et al., 2012). **(C)** A wireless inductive proximity sensor system designed for early detection of implant loosening by analyzing the electromagnetic field generated by passive sensors located around the implant. Reproduced with permission from (Mohammadbagherpoor et al., 2020). **(D)** Resistance sensors on the surface of the femoral head, with an image sensor to measure the position of the femoral head assembly relative to the acetabular assembly. Reproduced with permission from (Chen et al., 2015).

Some sensors can enhance intra-operative precision and aid surgeons in refining procedures. Chen et al. (2015) designed a visual-aided wireless monitoring system composed of two key components: the sensors and the display. The sensors include both contact and image sensors, enabling the estimation of the relative position between the femoral head and acetabular components. Manupibul et al. (2019) developed the Force-PRO device, which assists doctors in evaluating the force on the surface of the acetabular liner and the angle of the implant during surgery. The optimal angle reduces the risk of post-operative implant dislocation. Wijayaratna et al. (2021) designed a sensor attached to the hip prosthesis for measuring the pH of the joint fluid. When the pH decreases, it signals to the doctor that the hip prosthesis may be infected and requires prompt treatment. Recent advances in implantable hip sensors are detailed in Table 2, with representative studies highlighted in Figure 1.

**TABLE 2:** The applications of implantable sensors in hip.

Year	Author	Study	Classification	Conclusion
1999	Graichen et al.	CS	Resistive sensors	A hip endoprosthesis was instrumented with sensors to measure the joint contact forces and the temperature distribution along the entire length of the titanium implant
2000	Cristofolini et al.	CSS	Piezoelectric sensors	A miniature transducer was used to assess the cement–prosthesis interface forces in cemented devices
2004	Bergmann et al.	CS	Resistive sensors	Any impairment of such a mechanically balanced system will increase the musculoskeletal loads, and malposition of total hip implants or muscle deficits caused by the surgical approach must be avoided or minimized
2010	Damm et al.	CSS	Resistive sensors	A new instrumented hip joint prosthesis was developed which allows the *in vivo* measurement of the complete contact loads in the joint
2012	Bergmann et al.	CS	Resistive sensors	During the implantable sensor, peak forces are approximately twice as high during real stumbling as during any other activity and may range higher than eight-times the body weight
2019	Mohammadbagherpoor et al.	CSS	Inductive sensors	A wireless inductive proximity sensor system for detecting early implant loosening. The loosening of the implant is accurately detected by analyzing the electromagnetic field generated by the passive sensors located around the implant
2019	Manupibul et al.	CSS	Resistive sensors	An innovative Force-PRO device can aid the doctor in evaluating the force on the surface of the acetabular liner and the angle of the acetabular liner during the hip implant operation
2021	Heilemann et al.	CSS	Resistive sensors	Using eighteen sensors in positions across the acetabular bone-implant interface, micromotion magnitudes from 59 μm ± 2 μm–222 μm ± 5 μm were detected
2021	Wijayaratna et al.	CSS	Electrochemical sensors	An implantable sensor developed to measure synovial fluid pH for noninvasive early detection
2022	Wei et al.	CSS	Electric sensors	A new force measurement system was developed to provide surgeons with objective data to help determine the optimal implant fit and configuration

CS, clinical studies; AS, preclinical animal studies; CSS, cadaver specimen studies; PMHS, post-mortem human specimens.

#### 4.1.2 Knee

**FIGURE 2:**
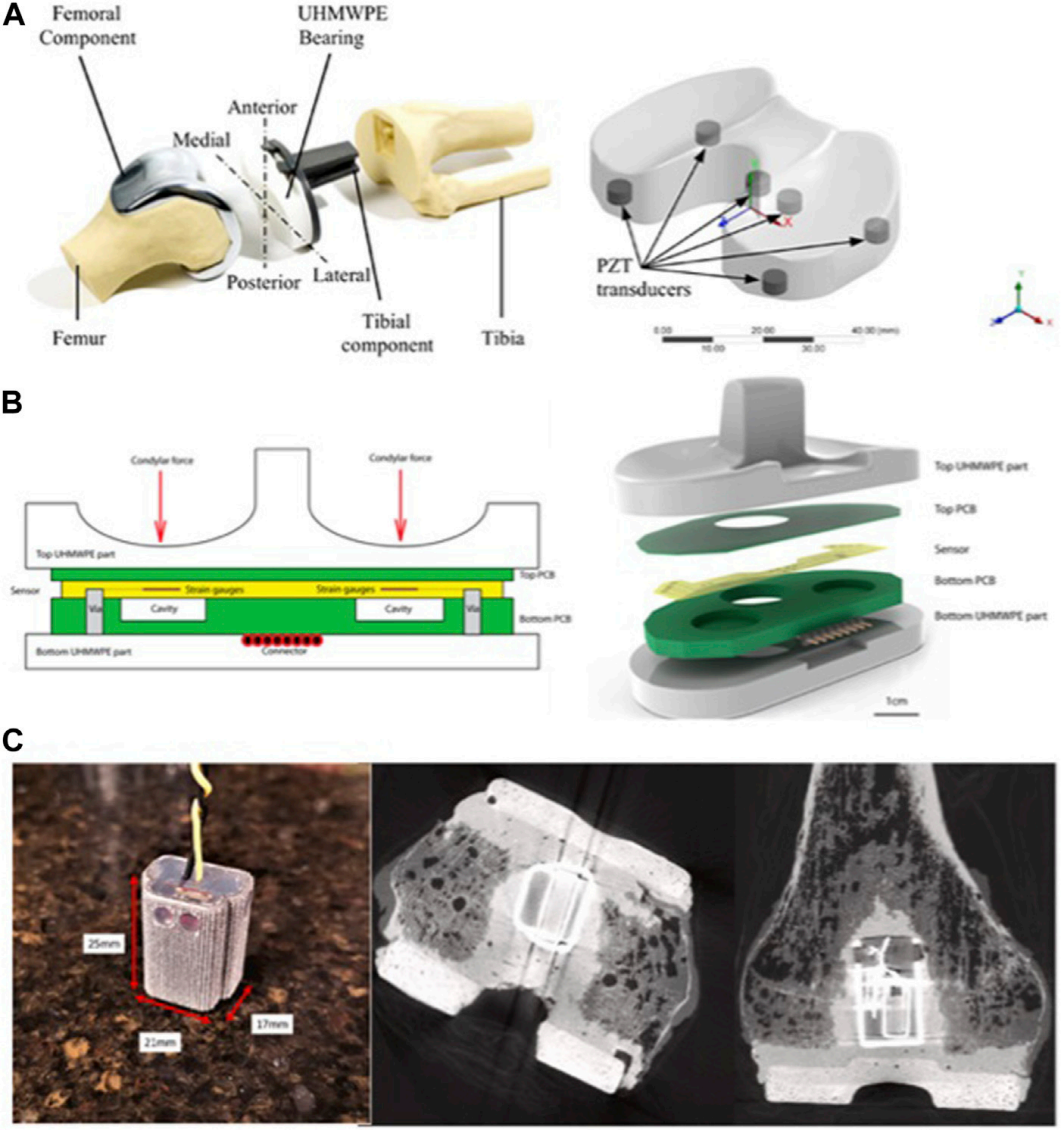
Implantable sensors for the knee: **(A)** A knee implant design with six piezoelectric transducers embedded in the tibial bearing, measuring total and compartmental forces and tracking contact points on the medial and lateral compartments of the bearing. Reproduced with permission from (Safaei et al., 2020). **(B)** A closed-structure sensor composed of printed circuit boards and a microfabricated polyimide thin-film piezoresistive strain sensor for each condylar compartment, to provide accurate tracking of slow and fast changing forces. Reproduced with permission from (Forchelet et al., 2014). **(C)** Sensor electronics embedded within a 3D printed titanium case and encapsulated with medical-grade silicone (left). The sensor is encased in bone cement and positioned in the intramedullary cavity of the femur (right). Reproduced with permission from (Lavdas et al., 2021).

Severe knee osteoarthritis can result in disability, significantly affecting quality of life. Several studies have demonstrated a significant link between knee joint forces and osteoarthritis (Amin et al., 2004; Niu et al., 2009). Therefore, understanding the forces acting on the knee joint following total knee arthroplasty (TKA) is crucial. These forces can impact the longevity of the implant and the integrity of the interface between the implant and the bone (Song et al., 2020). To gain insights into the mechanisms of knee disorders and post-operative knee care, direct measurement of the force in the knee joint can be achieved through implantable sensor technology. Recent advances in implantable sensors for knee applications are summarized in Table 3, with representative studies highlighted in Figure 2.

**TABLE 3:** The applications of implantable sensors in knee.

Year	Author	Study	Classification	Conclusion
2005	D’Lima et al.	CS	Resistive sensors	An electronic knee prosthesis was implanted to measure tibial forces *in vivo* during activities of daily living after TKA
2014	Forchelet et al.	CSS	Resistive sensors	The sensors can measure forces up to 1.5 times body weight with a sensitivity fitting the requirements for the proposed use and it has a good tracking of slow and fast changing forces in the knee prosthesis
2017	Safaei et al.	CSS	Piezoelectric sensors	A conceptual design of an UHMW knee bearing with embedded piezoelectric transducers was proposed, which was able to measure the reaction forces from knee motion as well as harvest energy to power embedded electronics
2018	Safaei et al.	CSS	Piezoelectric sensors	Promise for embedded piezoelectric transducers to create autonomous, self-powered *in vivo* knee implant force sensors
2020	Song et al.	CS	Resistive sensors	To evaluate intercompartmental load intraoperatively with a sensor after conventional gap balancing with a tensiometer during TKA
2020	MacDessi et al.	CS	Resistive sensors	Restoring the constitutional alignment with KA in TKA results in a statistically significant improvement in quantitative knee balance
2020	Safaei et al.	CSS	Piezoelectric sensors	An instrumented knee implant design with six piezoelectric transducers embedded in the tibial bearing to measure the total and compartmental forces as well as to track the location of contact points on the medial and lateral compartments of the bearing
2020	Vakiel et al.	CSS	Optical sensors	A novel, repeatable, and reliable method for measuring stress on the surface of articular cartilage in articular joints by FBG sensors
2021	Lavdas et al.	CSS	Resistive sensors	A telemetric sensor system to integrate with a bone cement spacer and measure knee joint temperature was designed and evaluated
2022	Sarpong et al.	CS	Resistive sensors	The use of a sensor-balancing device for soft tissue balancing in TKA did not confer any additional benefit in clinical outcomes, and it significantly increased operative time and costs associated

CS, clinical studies; AS, preclinical animal studies; CSS, cadaver specimen studies; UHMW, ultra high molecular weight; KA, kinematic alignment; TKA, total knee arthroplasty; FBG, fibre bragg grating.

In 1998, sensors were integrated into a distal femoral prosthesis to measure the forces acting on the femoral shaft in two patients throughout their daily activities for up to 2.5 years. Subsequently, these measurements were used to calculate the forces exerted on the knee joint (Taylor et al., 1998; Taylor and Walker, 2001). In 2004, the first direct *in vivo* measurement of knee forces was performed using a uniaxial force measurement device (D’Lima et al., 2005). However, this device could not measure anterior shear and axial torque, which led to the development of a new generation of knee sensors in 2005. The second-generation sensor design incorporated a redundant array of 12 strain gauges, collectively providing measurements for the three components of force and three moments (Kirking et al., 2006). In 2014, piezoelectric sensors were incorporated into knee implants, exploiting their self-powering capability (Strain gauge sensor made of PI) (Forchelet et al., 2014). In 2017, Safaei et al. (2017) introduced a knee bearing with four embedded piezoelectric transducers capable of measuring knee joint pressure (The piezoelectric transducer, diameter 8 mm, thickness 3 mm). They investigated the fatigue behavior of the bearing and the electromechanical performance of the embedded piezoelectric components (The piezoelectric transducer, diameter 8 mm, thickness 3 mm) (Safaei et al., 2017; Safaei et al., 2018; Ponder et al., 2019; Safaei et al., 2020). The findings revealed that a peak voltage of 3 V can be generated with a load resistor of 1 MΩ, and a peak average power of 5.5 μW can be obtained under a resistive load of 171 kΩ. Three preparation processes were also compared. Fused deposition modeling proved to be fast and cheap, but the results were not satisfactory. The longer the stereolithography print time, the higher the quality of the final product. Alternatively, computer numerical control machining provided superior material characteristics but was more labor intensive. Finally, they evaluated the distribution of piezoelectric sensors on the bearing using finite element analysis. The results indicated a maximum error rate of 2.6% for the device.

Implantable sensors are widely used to measure contact forces within joints during daily activities. Fregly et al. (2009) tested the medial contact force of the knee joint in a patient who had undergone knee arthroplasty and observed that, when the patient used a cane, this force was reduced by 27% and 16% compared with that when the patient was standing and walking without a cane, respectively. However, the benefits of using a cane were contingent on the side on which it was used. Walking with contralateral hand crutches resulted in a 43% reduction in knee contact force, while walking with ipsilateral hand crutches lead to an average increase of 9% (K-Scan sensors, Tekscan, South Boston, MA) (D’Lima et al., 2012).

In addition to monitoring everyday contact forces in the knee joint, implantable sensors can help doctors assess the condition around the prosthesis after TKA (MacDessi et al., 2020; Song et al., 2022b). Lavdas et al. (2021) implanted a sensor package into the femoral osteotomy of a cadaver (Temperature sensor, length 16.5 mm, width 23 mm). The sensor demonstrated an accuracy and precision of ± 0.24°C and 0.09°C, respectively. While additional testing is necessary for clinical application, the feasibility of this approach is evident. Sarpong et al. (2022) used sensors for soft tissue balancing during TKA, revealing that they did not provide additional benefits in clinical outcomes. Moreover, it significantly increased surgery time and incurred additional costs.

In recent years, researchers have focused on the development of multi-functional and smart implants. Yamomo et al. (2021) introduced a 3D-printed titanium interpositional device designed to integrate triboelectric generators into a commercially available TKA. The study evaluated the device’s stiffness, durability, and effectiveness as a housing for triboelectric generators.

Implantable sensors hold great promise for various commercial applications in the knee joint. Their integration into existing joint replacement components involves encapsulating the sensor, telemetry technology, and power supply system within the tibial tray, thereby increasing economic feasibility. A tibial tray integrated with sensors for the intra-operative monitoring of tibiofemoral force distribution and quantification of soft tissue balance enables the collection of extensive data on internal forces during the post-operative period. Surgeons find significant value in measuring *in vivo* forces and strains, while patients benefit by gaining insights into the forces within their knees, aiding in the identification of potentially risky activities.

#### 4.1.3 Shoulder

The shoulder is one of the most complex major joints in the body. Unlike the hip joint, the shoulder joint is characterized by exceptional flexibility and is primarily stabilized by muscles, while the hip joint is constrained by bony structures. Forces exerted in and around the shoulder are directly related to many common shoulder disorders, and some inappropriate forces generated during daily and sport activities can lead to shoulder injuries. Therefore, shoulder arthroplasty provides a convenient opportunity to implant sensors into the shoulder to monitor forces during complex activities. In 2007, Bergmann et al. (2007) pioneered the *in vivo* measurement of shoulder forces using sensors (Strain gauges, SG, type KSP 1-350-E4, Kyowa). To measure the contact force of the joint, six sensors were placed on the humeral stem during shoulder arthroplasty. Two years later, they conducted a comparative analysis of the contact forces in the shoulder joint during everyday activities using data from four patients with the aforementioned implants. The study revealed that the highest joint forces were recorded while steering a car with one hand, placing a 1.5 kg load on a table, and lifting a 2 kg weight to a high shelf (Westerhoff et al., 2009). Their findings strongly indicate that patients with shoulder problems or those recovering from shoulder surgery should avoid certain activities, such as lifting heavy objects with the arm outstretched. Furthermore, their research revealed that the frictional force within the shoulder joint exceeded previous estimates made using models that neglected its significance in shoulder calculations.

Reverse total shoulder arthroplasty (RTSA) is mainly applicable to cases of rotator cuff tear arthropathy that are not suitable for conventional total shoulder arthroplasty (TSA). However, the sensor designed by Bergmann et al. (2007) targets the TSA and may not be optimized for RTSA. Farmer et al. (2020) integrated four strain gauge sensors with a trial glenosphere to measure intra-operative glenohumeral contact forces during RTSA. Their findings indicate the feasibility of measuring joint contact forces intra-operatively in RTSA. Building on this, Leinauer et al. (2021) replaced the resistive sensors with capacitive sensors, citing their perceived benefits of enhanced stability and high sensitivity (Capacitive sensor made of PDMS). However, it is important to note that these sensors are intended solely for intra-operative measurements and must be removed before the procedure is completed. To date, there have been no studies that demonstrate the post-operative use of pressure sensors for shoulder measurements.

### 4.2 Spine

Sensor technology has served as a valuable tool in the exploration of spinal mechanics. As early as 1966, Waugh (1966) pioneered the use of Harrington rods equipped with strain gauges to measure *in vivo* forces during spine fixation. The strain gagues on the Harrington rods are connected to a machine via percutaneous wires and removed within a day. Subsequently, telemetry systems were used for early force measurements following spine surgery (Nachemson and Elfström, 1971; Elfström and Nachemson, 1973). Since then, the incorporation of strain gauges into cages and vertebral body replacement devices has significantly extended the duration of force measurements (Rohlmann et al., 1994; Szivek et al., 2005; Rohlmann et al., 2008). With the declining use of Harrington rods, a new generation of implantable sensors are now being positioned on the posterior side of the rod for spinal fixation. These sensors distribute the load across the spine and are used to measure forces in all directions (Cripton et al., 2000). While the measurement of the shared load of the spine can be captured through the posterior rod bar, it does not directly represent the forces acting on the spine itself. In contrast to systems with posterior rods, intervertebral and laminectomy implants (vertebral body replacements) are loaded in direct series with the spine, exposing them to the same forces as the spine (Ledet et al., 2000; Ledet et al., 2005; Rohlmann et al., 2010).

**FIGURE 3:**
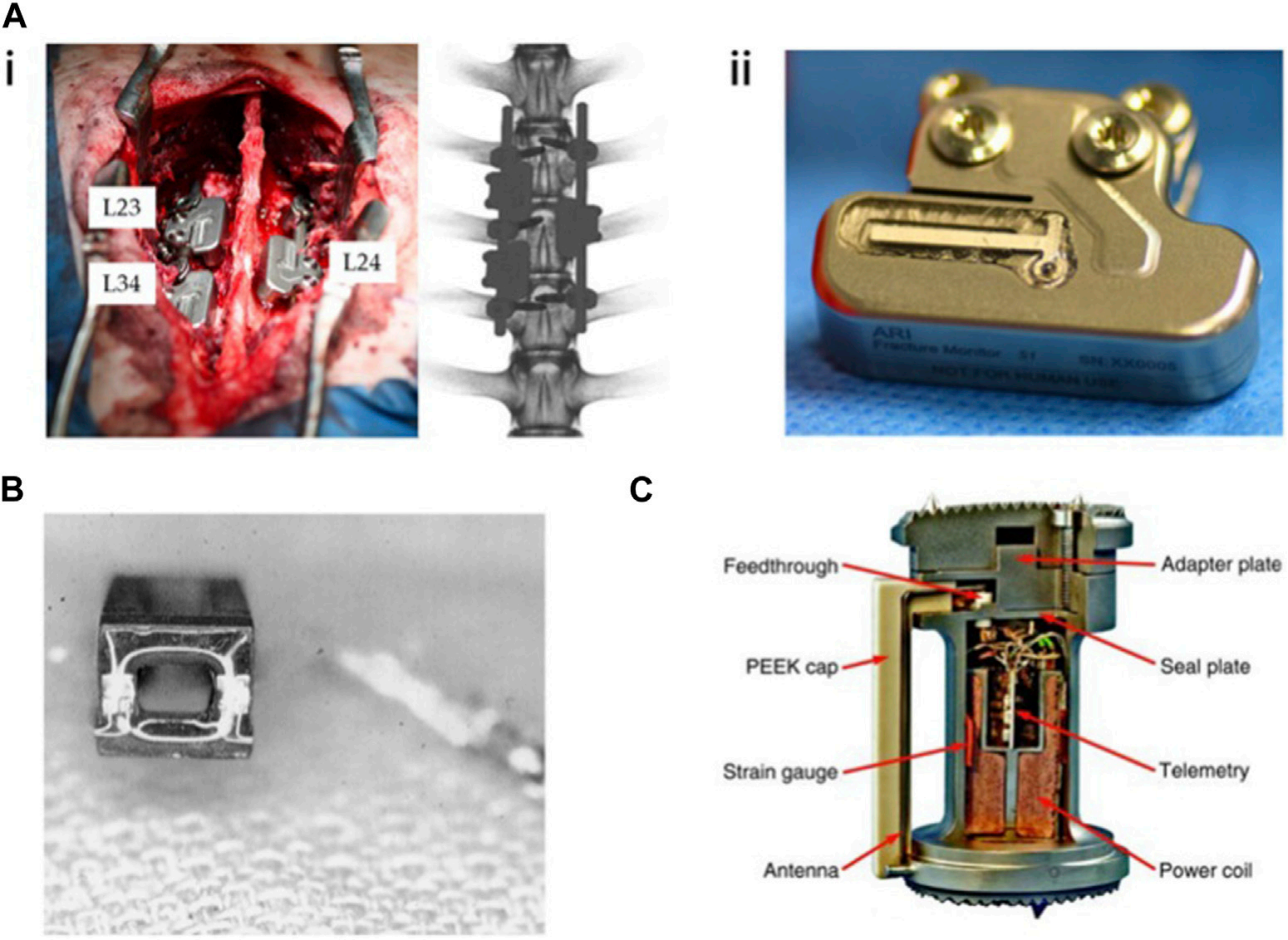
Implantable sensors for the spine: **(A)** (i) An implantable sensor system for continuous and wireless implant load monitoring following spinal fusion. (ii) Spine sensor prototype derived from an existing trauma sensor. Reproduced with permission from (Windolf et al., 2022a). **(B)** Strain gauges were mounted onto the cage and connected to a telemetry transmitter for real-time, *in vivo* spine load measurement. Reproduced with permission from (Ledet et al., 2000). **(C)** A vertebral body replacement with a strain gauge and six load sensors, integrated with an inductively powered implant and telemetry unit. Reproduced with permission from (Rohlmann et al., 2010).

The most important consideration following spinal fusion surgery is the determination of solid bone fusion. Recent studies propose that implantable sensor technology could serve as a quantitative means to assess fusion progression (Mularski et al., 2006). Windolf et al. (2022a) modified existing trauma sensors for integration with a standard pedicle screw-rod system, implanting them into a sheep’s body and collecting data with a smartphone. Through comparison with CT images, they concluded that implantable sensors have the capability to assess the progression of spinal fusion. Barri et al. (2022) introduced a self-powered sensing system designed to evaluate the process of spinal fusion (Fowler- Nordheim sensor). The system operates on the principle that during fusion, the modulus of elasticity in the fused portion of the spine increases, resulting in a decrease in strain on the fixation device. Several studies have explored disc pressure measurements using implantable sensors, primarily in animal studies (Pressure sensor die was selected, SM5112, Silicon Microstructures Inc., 2.0 mm × 2.0 mm×0.9 mm) (Glos et al., 2010; Roriz et al., 2014). Recent advances in implantable sensors for spinal applications are detailed in Table 4, with representative studies highlighted in Figure 3.

**TABLE 4:** The applications of implantable sensors in spine.

Year	Author	Study	Classification	Conclusion
1994	Rohlmann et al.	CSS	Resistive sensors	Telemetered AO spinal internal fixators were implanted in a patient with degenerative instability to determine the implant loads for different activities before and after additional anterior stabilization of the spine
2000	Cripton et al.	CSS	Resistive sensors	Intradiscal pressure and the forces and moments supported by the implants were measured using, respectively, a needle mounted pressure sensor and strain gauges mounted on the spinal implants
2005	Ledet et al.	AS	Resistive sensors	Instrumented interbody implants were placed into the disc space of a motion segment in two baboons to measure *in vivo* loads in the lumbar spine
2010	Mularski et al.	CSS	Resistive sensors	Continuous real-time visualization of individual vertebral body movements along the movement axes are possible with high accuracy using implantable microsensors
2010	Rohlmann et al.	CS	Resistive sensors	Six load sensors and a telemetry unit were integrated into the inductively powered implant. Three postures were studied: sitting freely, using a vertical backrest, and a backrest declined by an angle of 25°
2010	Glos et al.	CSS	Piezoelectric sensors	A compressive stress sensors packaged for extended, *in vivo* implantation in the annulus of the intervertebral disc
2014	Roriz et al.	AS	Optical sensors	Measure the intradiscal pressure signal of an anesthetized sheep under spontaneous breathing
2021	Glassman et al.	AS	Resistive sensors	A temperature sensing implant might reproducibly detect local temperature change associated with peri-implant wound infection, in a rabbit model
2022	Barri et al.	CSS	Piezoelectric sensors	A new self-powered sensing and data logging system for postoperative monitoring of spinal fusion progress
2022	Windolf et al.	AS	Resistive sensors	An implantable sensor system for continuous and wireless implant load monitoring after spinal fusion

CS, clinical studies; AS, preclinical animal studies; CSS, cadaver specimen studies.

Implantable sensors play an important role in spinal biomechanics by acting as essential measurement tools. They provide critical data that can optimize study models for clinicians and researchers, offering valuable insights for post-operative management. Moreover, they can serve as a diagnostic tool to determine if a spinal implant has become infected (strain gauges, REF: 179762120, Medicina 2022, Raynham, MA, United States) (Glassman et al., 2021). It is anticipated that implantable sensors will find widespread use as a quantitative diagnostic assessment tool in clinical settings in the future.

### 4.3 Fracture

**FIGURE 4:**
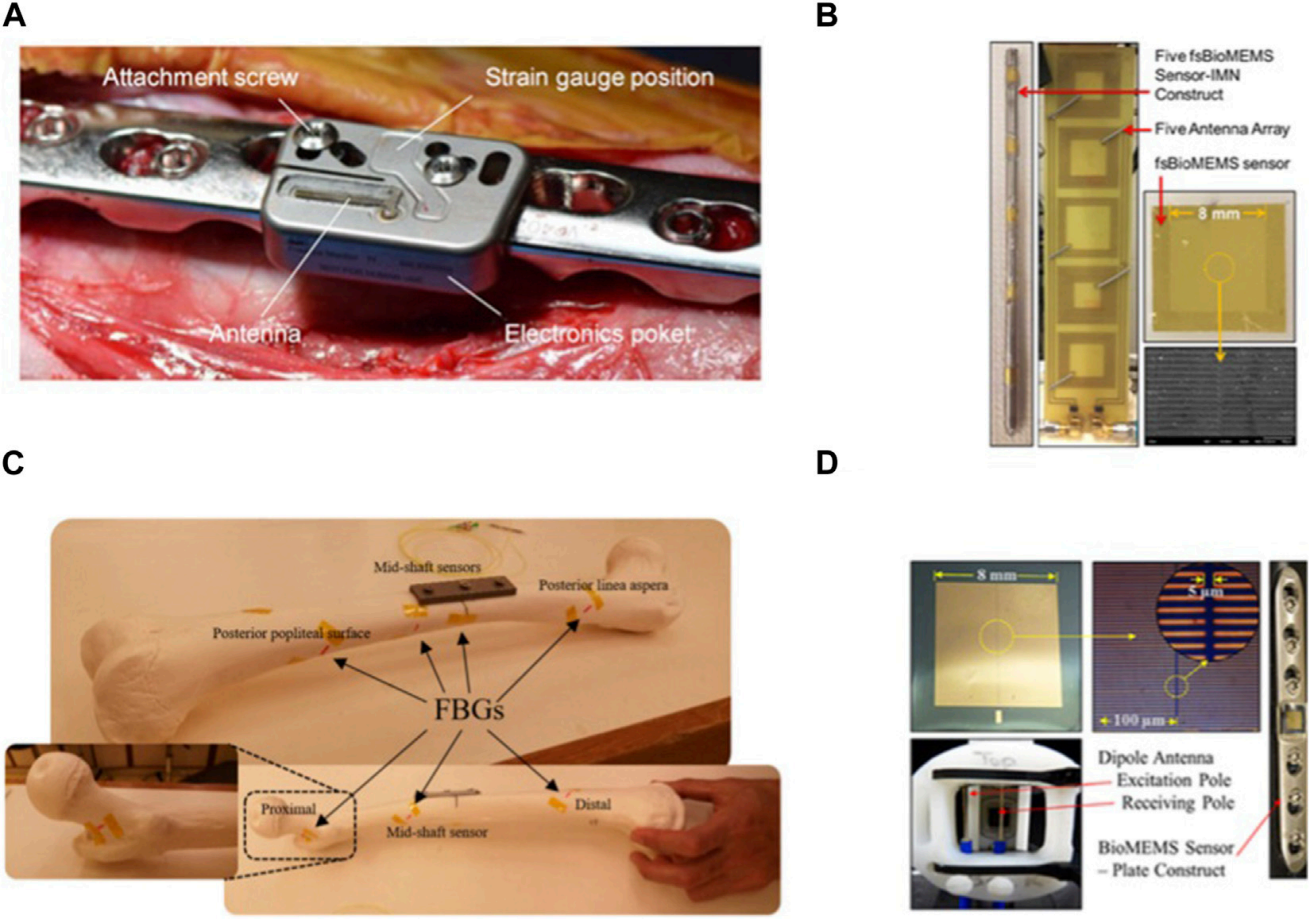
Implantable sensors for fractures: **(A)** An implantable sensor connected to a locking plate, capable of Bluetooth transmission to a smartphone. Reproduced with permission from (Windolf et al., 2022b). **(B)** Multiple implantable, flexible substrate, wireless, microelectromechanical (fsBioMEMS) sensors adhered to an intramedullary nail for quantifying the biomechanical environment along the length of fracture fixation hardware during simulated healing. Reproduced with permission from. Wolynski et al., 2019) **(C)** Plated femur sawbones equipped with seven fiber Bragg grating (FBG) strain sensors to monitor the femoral fracture healing process. Reproduced with permission from (Najafzadeh et al., 2020). **(D)** A wireless, biocompatible, implantable, microelectromechanical system (bioMEMS) sensor for diagnosing the *in vivo* course of fracture healing in the acute post-treatment period. Reproduced with permission from McGilvray et al., 2015).

Typically, fractures will consolidate within a few weeks following internal fixation. The speed and quality of healing are corelated with factors such as the fracture’s location and type, as well as the individual’s physical condition. Despite the increasing recognition in biomechanical research over recent decades regarding the important role of mechanics in fracture healing, some surgeons remain hesitant to implement early weight-bearing, fearing potential fixation failure and non-healing. Therefore, experts advocate for the assessment of fracture healing and the mechanical environment at the fracture site using sensor technology (McGilvray et al., 2015; Pelham et al., 2017; Klosterhoff et al., 2020). Recent advances in implantable sensors for fractures are detailed in Table 5, with representative studies outlined in Figure 4.

**TABLE 5:** The applications of implantable sensors in fracture.

Year	Author	Study	Classification	Conclusion
2015	McGilvray et al.	AS	Inductive sensors	An implanted sensor system assessing the bone healing status through continuous monitoring of the implant load
2017	Pelham et al.	CSS	Resistive sensors	The wavelength shifts and the corresponding strain sensitivities of the FBG sensors were measured to determine their effectiveness in monitoring the femoral fracture healing process
2019	Wolynski et al.	CSS	Inductive sensors	An implantable strain sensor platform and longitudinally measured strain across a bone defect in real-time throughout rehabilitation
2020	Klosterhoff et al.	AS	Resistive sensors	The simultaneous use of multiple implantable flexible substrate wireless microelectromechanical sensors adhered to an intramedullary nail to quantify the biomechanical environment along the length of fracture fixation hardware during simulated healing in *ex vivo* ovine tibiae
2020	Najafzade et al.	CSS	Optical sensors	Using FBG sensors to objectively measure fracture healing
2022	Windolf et al.	AS	Resistive sensors	The development and evaluation of a wireless, biocompatible, implantable, microelectromechanical system sensor, and its implementation in a large animal model

CS, clinical studies; AS, preclinical animal studies; CSS, cadaver specimen studies; FBG, fibre bragg grating.

In the early 1970s, Burny et al. (1984) used fracture plates equipped with strain gauges to measure loading during fracture healing via percutaneous wires. Subsequently, Brown et al. (1982) used battery-powered sensors to monitor forces in proximal femoral nail plate fixation systems. The results revealed an unexpectedly high axial force distance during walking in the early phase of fracture prognosis. An intramedullary femoral nail equipped with sensors was used to monitor femoral forces during fracture healing. The findings indicated a 50% decrease in load during the first 6 months following fixation (Schneider et al., 2001).

Currently, the assessment of fracture healing primarily relies on X-rays, which are highly dependent on the subjective judgment of the physician. X-rays also pose issues of radiation exposure and lack the capability for continuous assessment of the state of healing. Windolf et al. (2022b) developed a fracture monitoring system using implantable sensors. The sensor is positioned on an internally fixed plate and the data can be transmitted to a cell phone. Findings revealed that the sensor could autonomously function for 6.5–8.4 months until energy depletion. The implantation did not cause adverse effects such as immune rejection. Simultaneous monitoring of the load on the internal fixation correlated with the X-ray scoring range. Typically, a single sensor is placed on the internal fixation implant for monitoring. Wolynski et al. (2019) developed an implantable, flexible, substrate-based, wireless microelectromechanical sensor sensor is a square with 8 mm sides and 0.8 mm thickness with tape substrate, gold metal layering, and a Si3N4 dielectric layer. These sensors can be applied in multiples by adhering them to the intramedullary nail, enabling quantification of the biomechanical environment along the length of the fracture fixation hardware. Conceição et al. (2023b) introduced an ultra-sensitive capacitive sensing system designed for intelligent implantable fixation devices, capable of effectively monitoring the evolution of fractures. The outcomes of *in vitro* experimental testing and numerical simulations highlight the promising potential of capacitive technology. In addition to traditional strain gauge sensors, fiber Bragg grating (FBG) sensors are also used to monitor fracture healing. The high sensitivity of FBG sensors ensures accurate measurements, even under relatively small loads (Najafzadeh et al., 2020).

## 5 Current limitations and future perspectives

While implantable sensors have been under development for almost 50 years, certain limitations remain, such as chronic inflammation, infection, degradation, migration, and limited sensing capability. Inflammation is a normal response to foreign materials; however, despite the use of biocompatible materials in implantable sensors, managing inflammation remains a primary concern. Chronic inflammation can result in decreased peri-implant tissue loading, ultimately leading to implant failure or loosening. Therefore, identifying and addressing inflammation before serious harm occurs is crucial to preventing serious complications. Infection also poses a significant challenge for implantable sensors. Bacteria adhere to the sensor surface, creating a biofilm that prevents the action of antibodies, immune cells, and antibiotics. Once an infection occurs, it can lead to sepsis and death if left untreated. Therefore, the early detection of infection indicators is imperative for timely intervention. If necessary, removal of the implant and sensor should be considered. Implantable sensors are exposed to various physiological factors, leading to wear, corrosion, degradation, and potential fracture. Individual electronic component failure can also result in sensor malfunction while sensor fracture may release corrosion by-products, triggering inflammation. In such cases, early intervention is required for the removal of sensors and implants.

The development of next-generation sensors will incorporate flexible and biodegradable materials. New composites are continuously being developed, such as those composed of natural and synthetic polymers, resulting in mechanical properties close to those of natural tissues. Moreover, there is a demand for biomaterials with surfaces resistant to bacterial adhesion, aiming to mitigate the risk of infection. Advanced manufacturing techniques, coupled with the integration of 3D printing and other methods, are poised to facilitate the creation of personalized implants that incorporate sensors. Cells isolated from patients have the potential to be encapsulated into biomaterials to form bioink for the manufacture of 3D bioprinted structures. In the future, power supply systems will be optimized to meet the needs of increasingly complex implants. Harvesting energy from human activities and converting it into electrical energy for sensors is a promising and cost-effective approach. This method eliminates the need for frequent maintenance, ensuring sustainable and long-term energy generation. Multi-functional implantable sensors represent a popular research direction at present. These types of sensors not only offer feedback on traditional monitoring data but also have the potential to optimize treatment based on the obtained results. For example, a supply of antibiotics could be stored in a sensor that monitors infection and the targeted release of antibiotics could be strategically employed for early treatment, guided by changes in the monitoring data or doctor’s instructions. In addition, sensors dedicated to monitoring fracture healing could offer supplementary electrical stimulation to accelerate the fracture healing process.

## 6 Conclusion

This review first focuses on the classification of sensors and their operational principles, providing examples of their practical clinical applications. Subsequently, we explore the materials used in implantable sensor construction, offering a concise overview of traditional rigid materials and the emergence of flexible materials. Next, we describe in detail the application of implantable sensors in the musculoskeletal system, categorizing their use into three distinct areas: joints, spine, and fractures, depending on their specific application scenario. Finally, we discuss the limitations of current implantable sensors and outline future development trends. As sensor technology continues to advance and cross-disciplinary collaboration between medical and engineering fields grows, the use of implantable sensors in the musculoskeletal system is poised to become increasingly prevalent.
